# Adverse Effects of Non-Medical Use of Cannabis or Opioids Associated with Adverse Childhood Experiences

**DOI:** 10.3390/ijerph23050574

**Published:** 2026-04-29

**Authors:** Maria V. Aslam, Cherie Rooks-Peck, Curtis Florence, Sarah Beth L. Barnett, Claudia Gaffney, Elizabeth A. Swedo

**Affiliations:** 1 National Center for Injury Prevention and Control, Centers for Disease Control and Prevention, Atlanta, GA 30341, USA; 2 Department of Surgery, Emory Healthcare, Atlanta, GA 30322, USA

**Keywords:** ACEs, adverse childhood experiences, cannabis use, opioid use, cannabis use disorder, opioid use disorder

## Abstract

**Highlights:**

**Public health relevance—How does this work relate to a public health issue?**

- Non-medical use of cannabis or opioids and their adverse health effects represent growing public health concerns amid increasing cannabis legalization and the ongoing opioid overdose epidemic.
- Adverse childhood experiences (ACEs), a widespread and preventable public health problem, are strongly associated with substance use behaviors, yet their contribution to substance-related harms is not well quantified.

**Public health significance—Why is this work of significance to public health?**

- This study quantifies that nearly 65% of adverse health effects from non-medical cannabis or opioid use are attributable to exposure to two or more ACEs, highlighting a substantial and previously undermeasured burden.
- By linking ACEs to both substance use and resulting adverse health effects using population-level data, this work advances understanding beyond prior studies that focused primarily on use rather than harm.

**Public health implications—What are the key implications or messages for practitioners, policy makers and/or researchers in public health?**

- Preventing ACEs through evidence-based strategies (e.g., economic supports, early childhood interventions, and family-focused programs) may substantially reduce substance-related harms in adulthood.
- Integrated public health approaches that address both childhood adversity and substance use are critical for reducing long-term health consequences and improving population health outcomes.

**Abstract:**

Non-medical use of cannabis (NmC) and/or opioids (NmO) can lead to adverse health effects (AHEs), yet the proportion of these harms attributable to adverse childhood experiences (ACEs) remains unclear. This study estimated the contribution of ACEs to AHEs from NmC and/or NmO among adults aged ≥18 years using 2019–2020 Behavioral Risk Factor Surveillance System data from Arizona and Massachusetts. We conducted a retrospective cohort analysis of 24,739 respondents, linking past ACE exposure to self-reported NmC/NmO/NmC&NmO and related AHEs. Generalized linear models with a log link and binomial distribution adjusted for socio-demographic, healthcare access, and geographic factors were used to estimate associations and population-attributable fractions (PAFs). Propensity score methods matched respondents with and without ACEs on demographic and location characteristics. Among all the adults, 17.9% reported NmC, 5.8% reported NmO, and 2.4% reported NmC&NmO; among users of NmC/NmO/NmC&NmO, 5.0%/13.2%/36.0% reported AHEs. Among the respondents reporting AHEs from non-medical substance use, exposure to ≥2 ACEs was common (NmC: 89%; NmO: 82%; NmC&NmO: 84%). Compared to adults without ACEs, those with ≥2 ACEs had a higher likelihood of AHEs for NmC (adjusted relative risk [aRR] = 3.54, 95% CI: 1.65–7.59) and NmO (aRR = 3.64, 95% CI: 1.99–6.66) but not NmC&NmO (aRR: 1.86, 95% CI: 0.84–4.09). PAFs indicated that 63% (NmC) to 64% (NmO) of AHEs among the adults reporting NmC or NmO were attributable to ≥2 ACEs. Preventing childhood adversity may substantially reduce substance-related harms in adulthood.

## 1. Introduction

The legal treatment of cannabis use has undergone significant transformations in recent years, with 24 states enacting measures that permit adult non-medical use [1,2]. This shift in the cannabis policy landscape has coincided with the opioid overdose epidemic, during which the drug overdose deaths involving opioids increased five-fold from 2003 to 2023 [3]. Commonalities in non-medical use of cannabis (NmC) or opioids (NmO) can be attributed to several factors: cannabis may lead to use of non-prescription opioids [4,5,6,7], cannabis used concurrently with opioids may enhance analgesic opioid effects [8], cannabis may be used as self-treatment to alleviate opioid withdrawal symptoms [9,10], or adults may use cannabis to substitute for non-medical opioids use. The intricate relationship between these two substances necessitates research into the health implications when they are used separately and concurrently, as well as an understanding of risk factors that may be related to co-use-related harms, such as adverse childhood experiences (ACEs).

ACEs are potentially traumatic events that occur in childhood (0–17 years) [11]. A substantial body of literature has documented an increased risk of cannabis or illicit drug use among individuals with ACEs [7,12,13,14,15,16,17,18]. However, given that nearly 50% of U.S. states have already legalized the non-medical use of cannabis, it is now crucial to expand focus from risk factors for substance use to understanding risk factors for adverse health effects (AHEs). AHEs associated with non-medical cannabis and/or opioid use range in type and severity, including psychiatric and mental health effects [19,20,21,22]; substance use disorder [22,23]; withdrawal symptoms [22]; emergency room visits and hospitalizations [24,25,26]; acute poisoning and overdose [22,27]; and mortality [22,28,29,30]. Despite the scope and severity of AHEs associated with non-medical substance use [31], less is known about factors that modify risk for AHEs [32], including ACEs. Presently, evidence of the relationship between ACEs and substances’ AHEs comes predominantly from smaller studies that analyzed adults in a single healthcare facility [12] or adolescents in select school districts [7,18]. Among a few larger studies, some utilized data from over a decade ago, while others did not attempt to assess the proportion of AHEs attributable to ACEs [7,12,13,14,18]. To address the evolving landscape of cannabis use in the United States and the complex relationship between cannabis and opioid use, this study analyzed the recent data from Arizona and Massachusetts—two states that included detailed questions on AHEs due to NmC or NmO on their Behavioral Risk Factor Surveillance System (BRFSS) surveys. We estimated the prevalence of NmC, NmO, non-medical use of both substances (NmC&NmO), and AHEs from non-medical substance use; examined associations between cumulative ACE exposure, NmC/NmO/NmC&NmO, and AHEs; and calculated population-attributable fractions to estimate the proportion of AHEs due to one or both substances that could potentially be averted by preventing childhood adversities.

## 2. Materials and Methods

This analysis used individual-level 2019–2020 data from the BRFSS survey―an annual, random-digit-dialed cellular and landline phone survey collecting self-reported information on health conditions and risk behaviors from U.S. adults 18+ years who live in a private residence or college housing [33]. The median survey response rates in 2019/2020 were 49.4%/46.8%, respectively. As a secondary analysis of publicly available data without personal identifiers, this study did not require institutional review board approval.

### 2.1. Measures

#### 2.1.1. Exposures

For each 2019–2020 BRFSS respondent, individual-level ACE exposures (Table 1) were obtained through small area estimation (SAE). As described in Aslam et al. [34,35], predictive SAE models—logistic regressions (LR) and multilevel mixed-effects LR (MMEL) performed on each ACEs—controlled for individual-level BRFSS variables and state-level factors from five external sources, including socio-demographic characteristics, access to care, and ACE-related health outcomes. Model performance was evaluated by comparing observed ACE measures from BRFSS with corresponding predictions using standard metrics (e.g., sensitivity, specificity, predictive values, percent agreement, and Pearson correlation). Resampling methods—jackknifed MMEL/LR that leave out one state at a time—were used as sensitivity analyses, since they reduce possible state-specific non-response bias by resampling models with different ACEs non-response patterns. Additional details on model specification, validation performance, and limitations are provided in Aslam et al. [34,35]. SAE-based affirmative individual ACEs (household member with mental health or substance use problems, or incarcerated; parental separation/divorce; witnessing intimate partner violence; or experiencing physical, emotional, or sexual abuse during childhood) were summed and then grouped into exposure to no, any, or 2+ ACEs [36]. Coding of qualifying responses to each ACE question [37,38] is described elsewhere.

#### 2.1.2. Outcomes

For those 2019–2020 BRFSS survey participants who resided in Arizona or Massachusetts, we linked estimates of ACE exposure during childhood with the state-added individual-level data on the following six outcomes (Table 2) self-reported during adulthood:

1. Non-medical use of cannabis (NmC);
2. Non-medical use of opioids (NmO);
3. Non-medical use of both substances (NmC&NmO);
4. Adverse health effects due to non-medical use of cannabis alone (NmC AHEs);
5. Adverse health effects due to non-medical use of cannabis alone NmO alone (NmO AHEs);
6. Adverse health effects due to either of the two used substances (NmC&NmO AHEs).

#### 2.1.3. Covariates

Covariates (Table 1) included demographic characteristics (sex, race/ethnicity, and age), socio-economic characteristics (marital status, education, employment, annual household income, and home ownership), healthcare access (availability of health care coverage, access to a personal healthcare provider, and need to skip medical visits due to costs), and location (urbanicity and metropolitan statistical area [MSA] identifier).

### 2.2. Statistical Analysis

#### 2.2.1. Inclusion and Exclusion Criteria

Of those 26,141 survey respondents who received NmC/NmO questions in Arizona (N = 19,232) and Massachusetts (N = 6909), we excluded 1402 (5.36%) respondents who did not live in the state when surveyed, leaving 24,739 (94.64%) participants total, including 17,830 respondents in Arizona and 6909 respondents in Massachusetts (92.71% and 100.00% of the respondents who received NmC/NmO questions in each state, respectively). The non-response rate by outcome was 2.95% for NmC, 0.58% for NmC AHEs, 0.19% for NmO, 36.63% for NmO AHEs, 13.26% for NmC&NmO, and 0.29% for NmC&NmO AHEs.

#### 2.2.2. Propensity Score Matching

To better isolate the exposure effect, we used propensity score methods (Stata v17.0 StataCorp LP, College Station, TX, USA; command *psmatch2*) to match characteristics of persons with ACE exposure to persons without ACEs on the characteristics that were unlikely affected by ACE exposures, including location (state, urbanicity, MSA) and demographics (sex, race/ethnicity, age) [39,40]. Propensity score methods are particularly useful when randomization of exposure is unethical (e.g., intentional exposure of children to ACEs) [39]. To reduce bias and improve the precision of exposure estimates, we conducted a one-to-many match by applying kernel weights appropriate for complex survey designs [39,41] and using Mahalanobis distance between model covariates [42,43]. Among the six study outcomes, we matched the characteristics of persons with/without ACE exposure only for substance use (NmC, NmO, and NmC&NmO) and not for resulting AHEs, since AHE intensity may depend on epigenetic characteristics unobserved in our model [4].

In all substance use specifications, propensity score matching ensured a close match (Table S1) between demographic characteristics of the respondents with and without exposure to ACEs (zero standardized differences in covariates across the exposure and non-exposure groups; sample balance *t*-test *p*-value = 1.00). Similarity in demographic characteristics between the persons with and without ACE exposure allowed for minimizing the estimated impact of those characteristics on outcomes and ensuring that any differences in outcomes between persons with/without ACE exposure were likely attributable to the impact of ACEs rather than to the different distribution of covariates within the exposure and non-exposure groups [44].

#### 2.2.3. Modeling

We assessed the association between our six outcomes and exposure to any or 2+ ACEs by using the survey-weighted generalized linear models (GLMs) with log link and binomial distribution, where module-appropriate survey weights were adjusted for multi-year complex survey design [33,45], and standard errors were estimated using Taylor linearization (Stata v17.0 *vce(linearized)*) default. Each GLM model included one cumulative ACE variable at a time (any ACEs versus exposure to 2+ ACEs), with adjusted models additionally controlling for the respondents’ demographic and socio-economic characteristics, healthcare access, and location. Population-attributable fractions (PAFs) by ACE category were estimated for the outcome/ACEs combinations with statistically significant (two-sided *p* < 0.05) associations by using Miettinen’s method; PAFs reflect the proportion of prevalent conditions associated with ACE exposure under model assumptions and do not imply causality [46,47] (Stata v17.0 command *punaf* [48]). All analyses were performed from October 2023 to September 2024.

#### 2.2.4. Sensitivity Analyses

Additionally, we conducted two types of sensitivity analyses. While our main results combined Arizona’s and Massachusetts’ data into a pooled sample (Table 3); we also conducted separate analyses for Arizona and Massachusetts to evaluate state-specific differences in defining study outcomes (Table 4), with Arizona predominantly focusing on recent substance use (NmC AHEs: within past 6 months; NmO AHEs and NmC/NmO: past 12 months; Table 2) and short-term AHEs (e.g., anxiety, vomiting, and breathing problems after using a substance), while Massachusetts focused on lifetime use and AHEs related to substance use disorder (e.g., feeling addicted or experiencing trouble getting off a substance). Second, we used the 2020 BRFSS data from Arizona—a state and year where both the actual and predicted responses to ACEs questions were available [34,35]—and conducted separate analyses involving either the actual or predicted responses to assess the sensitivity of key findings to the use of predicted ACE estimates. In sensitivity analyses involving subgroups with smaller samples (e.g., AHEs to both substances in each state), we used two-sided *p* < 0.1 to account for the potential for type I error [49,50].

## 3. Results

**Figure 1:**
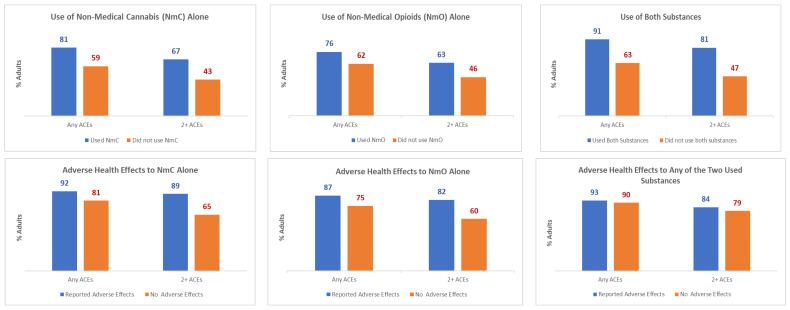
ACE Prevalence Among Adults Who Reported the Non-medical Use of Cannabis and Opioids and the Resulting Adverse Health Effects in Arizona and Massachusetts, 2019–2020. Weighted estimates of ACE prevalence are reported by substance use or adverse health effects category. For instance, the exposure to 2+ ACEs was estimated to be 67% among the adults in Arizona and Massachusetts who in 2019–2020 reported the non-medical use of cannabis (NmC) and 43% among the adults who did not report NmC.

In Massachusetts and Arizona, an estimated 1,565,367 (17.9%) adults reported the use of NmC; 515,525 (5.8%) adults reported NmO; and 222,731 (2.4%) reported NmC&NmO (Table S2). Of those, 78,797 adults (5.0% of NmC) reported NmC AHEs; 67,895 adults (13.2% of NmO) reported NmO AHEs; and 80,159 adults (36.0% of NmC&NmO) reported NmC&NmO AHEs. Among the adults who reported substance use and resultant AHEs, the percentage of adults with past exposure to ACEs was considerably higher compared to the adults with no reported substance use (Figure 1; Table S2). For instance, the exposure to 2+ ACEs was estimated to be 67%/63%/81% among the adults who reported NmC/NmO/NmC&NmO, respectively―a significantly (*p* < 0.001) higher percentage as compared to 43%/46%/47% of the adults who did not report the use of those substances. Among the adults with non-medical use of substances, the exposure to 2+ ACEs was estimated to be 89%/82%/84% among the adults who reported NmC/NmO/NmC&NmO AHEs, which was substantially higher as compared to 65%/60%/79% of the adults who did not report AHEs (*p*-value of <0.001 < 0.001/0.353, respectively).

Regression analysis corroborated the key associations between ACEs, non-medical use of substances, and resultant AHEs (Table 3). Compared to the adults without ACE exposures, the probability of reporting non-medical substance use was significantly higher for all outcomes and exposures (NmC, NmO, or NmC&NmO; any ACEs or 2+ ACEs). By contrast, the results for AHEs were statistically significant for each individual substance (but not for non-medical use of both substances) among the adults exposed to 2+ ACEs. Notably, compared to the adults without ACE exposure, among the adults exposed to 2+ ACEs, the probability of AHEs was approximately 250% higher for each type of non-medical substance use (NmC: aRR = 3.54, 95%CI, 1.65–7.59; NmO: aRR = 3.64, 95%CI, 1.99–6.66). PAFs for Massachusetts and Arizona (Table 3) demonstrate that preventing exposure to 2+ ACEs may contribute to averting one in three NmC or NmO (NmC: PAF = 32.38%, NmO: PAF = 36.17%) or one in two NmC&NmO (PAF = 53.96%). Furthermore, preventing exposure to 2+ ACEs may contribute to averting six in ten NmC AHEs or NmO AHEs (NmC PAF = 62.78%, NmO PAF = 64.10%).

The sensitivity analyses corroborated the main findings from this study. Regardless of the state-specific differences in defining study outcomes, we found consistently higher NmC or NmO and resulting AHEs among the adults with past exposure to 2+ ACEs (Table 4). For instance, compared to the adults without ACE exposure, the probability of NmC AHEs among the adults with past exposure to 2+ ACEs was 105% higher in Arizona, which examined short-term AHEs (aRR = 2.05, 90%CI, 1.04–4.02), and 608% higher in Massachusetts, which examined longer-term AHEs (aRR = 7.08, 95%CI, 2.76–18.15), while the probability of NmO AHEs was 143% higher in Arizona (short-term AH aRR = 2.43, 95%CI, 1.17–5.06) and 228% higher in Massachusetts (longer-term AHE aRR = 3.28, 95%CI, 1.06–10.22). The resulting PAFs demonstrated that preventing 2+ ACEs may contribute to averting 40–80% of NmC AHEs (Arizona short-term AHE PAF = 37.30%, Massachusetts longer-term AHE PAF = 79.14%) or 50–65% of NmO AHEs (Arizona short-term AHE PAF = 50.26%, Massachusetts longer-term AHE PAF = 64.57%).

Furthermore, the sensitivity analyses demonstrated robustness of key findings from this study to the use of the actual versus predicted ACE estimates. Due to the high accuracy of predicted ACE exposures—83%/100% of the respondents who actually experienced any/2+ ACEs were predicted to experience any/2+ ACEs—results from specifications that involved actual ACE exposure closely matched specifications with predicted ACE exposures (Supplementary Materials Table S3). For instance, for both NmC/NmO and resulting AHEs, the predicted ACEs aRR/PAFs either matched the actual aRR/PAFs or represented the conservative lower bound of the effect.

## 4. Discussion

Using recent data from states that included questions on non-medical use of cannabis/opioids and resultant adverse health effects, we estimated considerably higher probability of reporting non-medical use of and AHEs to these substances among adults with past ACE exposure as compared to adults without ACE exposures. Compared to the adults without ACE exposure, the probability of non-medical use of either cannabis or opioids was approximately 100% higher and the probability of AHEs from either substance was approximately 250% higher among the adults with past exposure to 2+ ACEs. Consequently, estimated PAFs demonstrated that preventing exposure to 2+ ACEs may contribute to averting up to nearly 40% of NmC or NmO and up to 65% of AHEs from either substance.

The general patterns for the associations between ACEs and substance use/AHEs were consistent across model specifications. At the same time, the magnitude of the effect differed by state, likely reflecting variations in survey measurement of substance use/AHEs and differences in NmC legalization. For instance, the probabilities of NmO and associated AHEs among the adults with ACEs were higher in Massachusetts compared to Arizona (e.g., for 2+ ACEs and NmO AHEs: aRR = 3.28 in Massachusetts, aRR = 2.43 in Arizona), possibly reflecting the lifetime orientation of substance use questions in Massachusetts versus the past-year questions used by Arizona. Similarly, for Massachusetts, we estimated a higher probability of NmC compared to Arizona among people with ACEs (e.g., for 2+ ACEs: aRR = 2.02 in Massachusetts, aRR = 1.60 in Arizona), which may reflect either timing of NmC legalization (Massachusetts: 2016 [2,51]; Arizona: November 2020 [52,53] or duration of experiences (Massachusetts: lifetime use; Arizona: use within past 6 months). In terms of AHEs, Massachusetts included in their BRFSS a comprehensive list of questions to capture the signs of substance use disorder [54] due to NmC and focused on lifetime AHE experiences. By contrast, Arizona included questions on short-term AHEs related to substance use and focused on the use within the past 6 months. Consequently, the probability of NmC AHEs and related PAFs was consistently higher in Massachusetts as compared to Arizona (e.g., for 2+ ACEs: aRR = 7.08 in Massachusetts, aRR = 2.05 in Arizona). Although the data from these two states did not allow for exploring which of the reasons―either the definition of substance use/AHEs or the duration of experiences―contributed the most to higher aRR and PAFs in Massachusetts versus Arizona, we found consistently higher NmC/NmO use and related AHEs among the adults with past ACE exposure, regardless of state-specific variations in measurements. Furthermore, compared to the adults without ACE exposure, we estimated consistently higher probability of non-medical use of each substance and resultant AHEs among the adults with past ACE exposure, regardless of whether we analyzed non-medical use of cannabis or opioids. In terms of co-use, ACEs were consistently associated with increased use of both substances, regardless of whether we combined Arizona and Massachusetts or estimated them separately. The mixed results for NmC&NmO AHEs—the statistically insignificant results for Massachusetts alone or when combined with Arizona—underscore the need for additional longitudinal or quasi-experimental analyses to assess the changes in co-use AHEs after states legalized NmC, particularly among adults facing barriers to opioid treatment [55].

Direct comparisons of our results to a wealth of research on substance use are difficult, given heterogeneous definitions of ACEs and substance use [56,57]. Generally, our study corroborates previous findings demonstrating strong associations between ACEs and substance use [7,12,13,14,15,16,17,57,58,59,60,61]. Our study uniquely contributes to the literature by focusing on AHEs due to substance use and quantifying the proportion of NmC/NmO AHEs that could be averted by preventing ACEs, measured as PAF. While our cross-sectional study design limits causal inference of PAFs, our PAF estimates quantify the proportion of outcome burden in the population statistically associated with ACEs and therefore indicate the potential public health impact if those ACE exposures were reduced. Few studies have examined ACEs PAFs for substance use and AHE outcomes. Dube et al. used data from the original CDC-Kaiser study to estimate PAFs due to experiencing any ACEs: 63% for ever being addicted to illicit drugs and 64% for ever using injection drugs [12]. Similarly, a study by Swedo et al. among adolescents in a rural county in Ohio found that 71.6% of past 30-day use of NmO was attributable to experiencing any ACEs [7]. Consistent with Dube et al. and Swedo et al., we estimated PAF for NmO AHEs to be 64.10%. However, to our knowledge, our study is the first to estimate PAF for NmC AHEs (62.78%) and, equally noteworthy, to establish its comparability with the PAF for NmO AHEs (64.10%). Furthermore, in our study, the unique set of AHE questions asked by each state allowed for drawing conclusions about associations between ACEs and both short- and long-term AHEs due to NmC/NmO. We found considerable PAFs for experiencing 2+ ACEs and both short-term AHEs due to NmC (37.30%) or NmO (50.26%) in Arizona, and long-term AHEs due to NmC (79.14%) or NmO (64.57%) in Massachusetts. Elevated substance AHEs among adults exposed to ACEs may reflect various biological and environmental pathways, including genetic transmission of drug vulnerability, in utero drug exposure among adults with familial substance abuse [62], substance abuse as a coping mechanism for neurotransmitter dysregulation from ACEs-related stress [63], or pain management necessitated by increased reports of chronic pain among the adults who experienced child abuse/neglect [13]. Regardless of the reasons, the sizable PAFs―for various ACEs/AHE combinations estimated in our paper―highlight the strong potential for ACEs prevention as a public health tool to prevent substance use and resulting negative health consequences.

The interrelated, preventable problems of ACEs and substance use require a coordinated approach. Focusing on prevention of both ACEs and substance use through system-level changes, public education, and implementation of evidence-based policies/programs can prevent ACEs [64], mitigate their harms, and decrease both substance use and their AHEs [65]. The American Public Health Association (APHA) and CDC collaborated on the *Urgent. Related. Preventable.* initiative to highlight the need for a coordinated approach to address the interrelated crises of exposure to ACEs, overdose, and suicide [66]. Prevention strategies can be amplified through a comprehensive public approach to these problems, and through alignment of policy, funding, and programs to address both ACEs and substance use together, rather than focusing on the issues independently. Effective approaches to prevent ACEs include providing long-term social and economic supports, including paid family/sick leave, high-quality childcare, housing support [64], or early childhood home visitation [49]. Improving access to substance use treatment increases safe, stable, nurturing relationships/environments; mitigates the negative impact of ACEs for people with substance use disorder; and prevents ACEs in the next generation [64]. While our study quantifies the strong potential impact of ACEs prevention on use of and AHEs from NmC/NmO, additional research is needed to develop and translate evidence-based strategies that address the interconnectedness between ACEs and substance use prevention.

### Limitations

This study has several limitations. First, BRFSS relies on self-reported data that may be subject to recall bias [67,68,69,70]. Second, BRFSS data are collected for private residences and exclude populations at higher risk for NmC/NmO use and AHEs (e.g., those incarcerated or homeless). Third, NmO AHE non-response of 36.63% may introduce bias, with current estimates representing the upper bound of the effect if non-respondents did not experience AHEs and the lower bound otherwise. Fourth, ACE estimates for Arizona (2019) and Massachusetts (2019/2020) were predicted through small area estimation. Fifth, the results from two states are not generalizable nationwide.

## 5. Conclusions

Up to 40% of NmC or NmO and up to 65% of the resulting AHEs were associated with past exposure to 2+ ACEs. Upstream public health prevention strategies across sectors aimed to prevent ACEs [71,72,73] may decrease non-medical substance use and limit substance-related adverse health effects in the future.

## Figures and Tables

**Table 1:** Definition of study variables.

Variables	Definition
**Outcomes**	
Non-medical use of cannabis and/or opioids; resulting adverse health effects	Non-medical use of cannabis (NmC), opioids (NmO), or both substances (NmC&NmO). ^a^ Additional details provided in Table 2.
Adverse health effects from non-medical use of cannabis and/or opioids	Resultant adverse health effects (AHEs) due to NmC alone (NmC AHEs), NmO alone (NmO AHEs), or any of the two used substances (NmC&NmO AHEs). ^a^ Additional details provided in Table 2.
**Exposure**	
Adverse childhood experiences (ACEs)	Exposure to any or 2+ of the following ACEs: presence of household member with mental health or substance use problem, or incarceration; parental separation or divorce; witnessing intimate partner violence; or experiencing physical, emotional, or sexual abuse during childhood. ^b^
**Individual-level covariates**	
Demographic characteristics	
Sex	Male/female identifier
Race/ethnicity	Race/ethnicity identifier; categorized as White (non-Hispanic), African American (non-Hispanic), Asian (non-Hispanic), American Indian or Alaskan Native (non-Hispanic), Hispanic, other
Age, years	Respondent’s age in years; categorized as 18–34, 35–54, and 55 years or older
Socio-economic characteristics	
Marital status	Marital status identifier; categorized as married, divorced, widowed, separated, never married, member of an unmarried couple, missing/invalid
Education	Respondent’s education identifier; categorized as did not graduate from high school, graduated from high school, or attended college or graduated from college
Annual household income from all sources, USD	Annual household income identifier; categorized as less than $25,000 or missing, $25,000–$50,000, and $50,000 or more
Employment status	Employment status identifier; categorized as employed for wage, self-employed/homemaker/student, out of work, retired, unable to work, missing/invalid
Home ownership	Home ownership identifier; categorized as own, rent, other arrangements, or missing/invalid
Healthcare Access	
Access to a personal healthcare provider	Access to a personal healthcare provider identifier; categorized as yes (access to one or more providers) or no
Any health care coverage	Identifier for any kind of health care coverage, including health insurance, prepaid plans such as Health Maintenance Organizations, or government plans such as Medicare, or Indian Health Service Health care coverage identifier; categorized as yes or no
Skipped medical visits due to the cost	Identifier for respondents who had to skip medical visits within the past 12 months due to inability to cover medical costs; categorized as yes or no
Location	
Metropolitan statistical area (MSA) status	MSA identifier; categorized as metropolitan/non-metropolitan areas
Urbanicity	Identifier for urban/rural counties

Source: Centers for Disease Control and Prevention. Behavioral Risk Factor Surveillance System (BRFSS). Survey Data and Documentation. Available from https://www.cdc.gov/brfss/data_documentation/index.htm. (accessed on 21 April 2026); ^a^ Detailed definition of each outcome is provided in Table 2. ^b^ BRFSS ACEs questions and coding of qualifying responses to each ACEs question were previously described in the literature [34,35,37].

**Table 2:** Questions on the non-medical use of cannabis or opioids and resultant adverse health effects in the 2019–2020 Behavioral Risk Factor Surveillance System surveys.

Outcome	Survey Question	Qualifying Responses
Cannabis		
Non-medical use of cannabis	Massachusetts	
	Have you used Marijuana six or more times for non-medical purposes?	Yes, past year; Yes, more than a year ago
	Arizona	
	In the past year, have you ever used marijuana or hashish?	Yes, for non-medical use
Adverse health effects from non-medical use of cannabis	Massachusetts	
	Have you felt addicted to Marijuana or experienced trouble getting off marijuana that you used non-medically?	Yes, past year; Yes, more than a year ago
	Or affirmative answer to any of the following questions:	
	From your non-medical use of Marijuana, did you ever have symptoms of drug withdrawal after stopping use, such as: depression, sweating, yawning, or insomnia when you stopped using a drug? Or did you use the drug or a similar drug to relieve or avoid withdrawal symptoms?	Yes
	Did you often have days when you ended up using Marijuana/Hashish a lot more or for a much longer time than you intended?	Yes
	Have you often thought that you should quit or cut down on your Marijuana use, or tried to do so more than once, but without success?	Yes
	Have you ever felt such a strong desire or urge to use Marijuana that you could not resist it or could not think of anything else?	Yes
	Did your use of Marijuana often interfere with your responsibilities at home or with children, at work, or at school?	Yes
	Have you gone to an emergency room or obtained medical treatment as a consequence of your Marijuana use?	Yes
	Were there times in the past year when you were under the influence of Marijuana in situations where it could cause you or others harm? For example when you were driving a car?	Yes
	Arizona	
	(Conditional on non-medical use) During the past 6 months, have you experienced adverse health effects after using marijuana (such as anxiety, panic, nausea, vomiting, breathing problems)?	Yes
Opioids		
Non-medical use of opioids	Massachusetts	
	Have you taken pain killers such as Vicodin, Percocet, Darvon, Codeine, Morphine or OxyContin six or more times for non-medical purposes?	Yes, past year; Yes, more than a year ago
	OR	
	Have you taken Heroin or Fentanyl six or more times for non-medical purposes?	Yes, past year; Yes, more than a year ago
	Arizona	
	The last time you filled a prescription for pain medication in the past year, did you use any of the pain medication more frequently or in higher doses than directed by a doctor?	Yes
	What did you do with the leftover prescription pain medication?	Kept it; Used it for another unrelated pain/other purpose
	In the past year, did you use a prescription pain medication that was not prescribed specifically for you by a doctor, dentist, nurse practitioner, or healthcare providers?	Yes
Adverse health effects from non-medical use of opioids	Massachusetts	
	Have you felt addicted or experienced trouble getting off of that drug you used non-medically?	Yes, past year; Yes, more than a year ago
	Or affirmative answer to any of the following questions:	
	From your non-medical use of any Opioid such as Vicodin, Percocet, Darvon, Codeine, OxyContin, Heroin, or Fentanyl, did you ever have symptoms of drug withdrawal after stopping use, such as: depression, sweating, yawning, or insomnia when you stopped using a drug? Or did you use the drug or a similar drug to relieve or avoid withdrawal symptoms?	*Yes*
	Did you often have days when you ended up using a drug a lot more or for a much longer time than you intended?	Yes
	Have you often thought that you should quit or cut down on your drug use, or tried to do so more than once, but without success?	Yes
	Have you ever felt such a strong desire or urge to use a drug that you could not resist it or could not think of anything else?	Yes
	Did your use of a drug often interfere with your responsibilities at home or with children, at work, or at school?	Yes
	Have you gone to an emergency room or obtained medical treatment as a consequence of your Opioid drug use?	Yes
	Arizona	
	What was the main reason you used the medication differently than prescribed?	Prescribed dose did not relieve pain; to relieve other physical symptoms; to relieve anxiety or depression; to prevent or relieve withdrawal symptoms
	(Conditional on any of the qualifying answers for non-medical use of drugs) In the past year have you felt dependent on prescription pain medication or experienced trouble getting off of the medication when you no longer needed it for medical reasons?	Yes

Data source: 2019–2020 Behavioral Risk Factor Surveillance System (BRFSS) for the states of Arizona and Massachusetts. BRFSS Questionnaires for Arizona are available from https://www.azdhs.gov/preparedness/public-health-statistics/behavioral-risk-factor-surveillance/index.php#questionnaires (accessed on 21 April 2026). BRFSS Questionnaires for Massachusetts are available from https://www.mass.gov/lists/behavioral-risk-factor-surveillance-brfss-questionnaires (accessed on 21 April 2026).

**Table 3:** Associations between the exposure to adverse childhood experiences and non-medical use of cannabis or opioids among adults in Arizona and Massachusetts in 2019–2020: relative risks and population-attributable fractions by ACE category.

	Adults by Outcome,	Any ACEs			2+ ACEs		
		Unadjusted RR	Adjusted RR ^a^	PAF, % ^b^	Unadjusted RR	Adjusted RR ^a^	PAF, % ^b^
	N (%)	(95% CI)	(95% CI)	(95% CI)	(95% CI)	(95% CI)	(95% CI)
**Substance use**							
Non-medical cannabis (NmC) alone	1,565,367 (17.88)	2.04 (1.67–2.49)	1.94 (1.66–2.25)	35.06 (26.76–42.41)	2.00 (1.73–2.32)	1.90 (1.68–2.16)	32.38 (25.16–38.90)
Non-medical opioids (NmO) alone	515,525 (5.76)	2.03 (1.67–2.45)	2.02 (1.66–2.46)	33.77 (24.62–41.81)	2.13 (1.80–2.53)	2.13 (1.79–2.53)	36.17 (28.28–43.19)
Both substances	222,731 (2.42)	5.74 (3.17–10.40)	4.36 (2.39–7.97)	65.63 (42.85–79.33)	3.82 (2.47–5.92)	3.12 (2.04–4.78)	53.96 (35.37–67.17)
**Adverse health effects from using one or more substances**							
Non-medical cannabis (NmC) alone	78,797 (5.03) ^c^	2.28 (0.98–5.26)	1.93 (0.81–4.62)	NA ^b^	3.53 (1.62–7.68)	3.54 (1.65–7.59)	62.78 (27.97–80.77)
Non-medical opioids (NmO) alone	67,895 (13.17) ^c^	2.67 (1.37–5.21)	2.16 (1.13–4.12)	45.37 (5.66–68.37)	3.72 (2.13–6.49)	3.64 (1.99–6.66)	64.1 (38.82–78.94)
Both substances	80,159 (35.99) ^c^	2.24 (1.09–4.63)	1.86 (0.84–4.09)	NA ^b^	1.32 (0.64–2.71)	1.34 (0.68–2.65)	NA ^b^

Abbreviations: ACEs = adverse childhood experiences; RRs = relative risks; PAF = population-attributable fraction. All the estimates in this table are weighted. For every substance use outcome, we used propensity score methods to better isolate the exposure effect and to match persons with ACE exposure to persons without ACEs on the characteristics that were unlikely to be affected by ACE exposures, including location (state, urbanicity, and MSA) and demographics (sex, race/ethnicity, and age). ^a^ Adjusted for the respondents’ demographic characteristics (sex, race/ethnicity, and age), socio-economic characteristics (annual household income from all sources, education, employment, marital status, and home ownership), access to healthcare (access to a personal healthcare provider and availability of any healthcare coverage), and location (metropolitan statistical area and urbanicity). ^b^ PAFs by ACE category were estimated for the outcome/ACEs combinations with statistically significant (two-sided *p* < 0.05) associations by using Miettinen’s method. ^c^ Percent of the adults who reported substance use.

**Table 4:** Associations between the exposure to two and more adverse childhood experiences and non-medical use of cannabis or opioids among adults in Arizona and Massachusetts in 2019–2020: relative risks and population-attributable fractions by state.

	Arizona				Massachusetts			
	(Past Year Use) ^a^				(Lifetime Use of Six or More Times)			
	Adults by Outcome,	Unadjusted RR	Adjusted RR ^b^	PAF, % ^c^	Adults by Outcome,	Unadjusted RR	Adjusted RR ^b^	PAF, % ^c^
	N (%)	(95% CI)	(95% CI)	(95% CI)	N (%)	(95% CI)	(95% CI)	(95% CI)
**Substance use**								
Non-medical cannabis (NmC) alone	323,852 (8.19)	1.88 (1.50–2.36)	1.60 (1.28–2.01)	24.51 (12.71–34.72)	1,241,515 (25.85)	2.05 (1.73–2.43)	2.02 (1.74–2.34)	33.89 (27.11–40.03)
Non-medical opioids (NmO) alone	446,568 (10.84)	2.00 (1.70–2.37)	1.92 (1.62–2.28)	32.19 (24.13–39.40)	68,957 (1.43)	3.04 (1.64–5.64)	2.86 (1.54–5.33)	48.89 (18.82–67.82)
Both substances	81,331 (1.93)	5.15 (3.05–8.71)	4.25 (2.54–7.11)	64.23 (44.89–76.82)	141,400 (2.83)	3.32 (2.07–5.31)	2.69 (1.70–4.26)	48.23 (26.39–63.59)
**Adverse health effects from using one or more substances**								
Non-medical cannabis (NmC) alone ^d^	17,149 (5.30) ^e^	1.33 (0.57–3.06)	2.05 (1.04–4.02)	37.3 (3.21–61.90)	61,648 (4.97)	5.85 (2.33–14.67)	7.08 (2.76–18.15)	79.14 (50.30–91.24)
Non-medical opioids (NmO) alone	38,558 (8.63) ^e^	2.81 (1.66–4.76)	2.43 (1.17–5.06)	50.26 (8.57–72.93)	29,337 (42.54)	4.30 (1.68–10.99)	3.28 (1.06–10.22)	64.57 (0.51–87.51)
Both substances ^d^	20,965 (25.78) ^e^	4.88 (1.18–20.19)	5.26 (1.07–25.88)	77.93 (0.03–95.13)	59,194 (41.86)	1.14 (0.62–2.11)	1.20 (0.67–2.16)	NA ^c^

Abbreviations: RRs = relative risks; PAFs = population-attributable fractions. All the estimates in this table are weighted. For every substance use outcome, we used propensity score methods to better isolate the exposure effect and to match persons with ACE exposure to persons without ACEs on the characteristics that were unlikely to be affected by ACE exposures, including location (state, urbanicity, and MSA) and demographics (sex, race/ethnicity, and age). ^a^ Arizona respondents who reported the non-medical use of cannabis were asked about the adverse effects that occurred within the past 6 months (rather than past year) of substance use. ^b^ Adjusted for the respondents’ demographic characteristics (sex, race/ethnicity, and age), socio-economic characteristics (annual household income from all sources, education, employment, marital status, and home ownership), access to healthcare (access to a personal healthcare provider and availability of any healthcare coverage), and location (metropolitan statistical area and urbanicity). ^c^ PAFs by ACE category were estimated for the outcome/ACEs combinations with statistically significant (two-sided *p* < 0.05) associations by using Miettinen’s method. ^d^ For NmC AHEs alone (Arizona) and for AHEs due to both substances (Arizona or Massachusetts), the results are shown for the two-sided *p* < 0.1 to account for the potential for type I error in the subgroup analyses with smaller sample sizes. ^e^ Percent of the adults who reported substance use.

## Data Availability

Restrictions apply to the availability of these data. Data were obtained from the Massachusetts Department of Public Health and the Arizona Department of Health Services and are available by contacting the states’ BRFSS coordinators.

## Supplementary Materials

The following supporting information can be downloaded at https://www.mdpi.com/article/10.3390/ijerph23050574/s1, Table S1: Socio-demographic characteristics of BRFSS respondents matched on exposure to ACEs; Table S2: ACE prevalence among the adults who reported non-medical use of cannabis and opioids and the resulting adverse health effects in Arizona and Massachusetts, 2019–2020; Table S3: Non-medical use of cannabis and opioids in Arizona in 2020: relative risks and population-attributable fractions for the actual ACEs compared to predicted ACE Exposures.

## Abbreviations

The following abbreviations are used in this manuscript:

ACEs	Adverse Childhood Experiences
NmC	Non-medical Cannabis
NmO	Non-medical Opioids
NmC&NmO	Non-medical Use of Both Cannabis and Opioids
AHEs	Adverse Health Effects
BRFSS	Behavioral Risk Factor Surveillance System
MSA	Metropolitan Statistical Area
PAF	Population-Attributable Fraction
GLM	Generalized Linear Model
aRR	Adjusted Relative Risk
CI	Confidence Interval
HMO	Health Maintenance Organization
CDC	Centers for Disease Control and Prevention
