# Supplementary material for: Adverse Effects of Non-Medical Use of Cannabis or Opioids Associated with Adverse Childhood Experiences
Source: Int J Environ Res Public Health. 2026 Apr 29;23(5):574. doi: 10.3390/ijerph23050574 (PMC13206216; doi:10.3390/ijerph23050574)
Supplement: Supplementary file 1 [file ijerph-23-00574-s001.zip › ijerph-4249963-supplementary.pdf]

Supplemental Online Content

Table S1: Socio-demographic Characteristics of BRFSS Respondents Matched on Exposure to ACEs.

Table S2: ACEs Prevalence Among Adults Who Reported Non-medical Use of Cannabis and Opioids and the Resulting Adverse Health Effects in Arizona and Massachusetts, 2019-2020.

Table S3: Non-medical Use of Cannabis and Opioids in Arizona in 2020: Relative Risks and Population Attributable Fraction for the Actual ACEs Compared to Predicted ACEs Exposures.

This supplemental material has been provided by the authors to give readers additional information about their work.

Table S1: Socio-demographic Characteristics of BRFSS Respondents Matched on Exposure to ACEs.

	Respondents Matched on Exposure to any ACEs										
	Respondents Who Reported										
	Non-medical Use of Cannabis (NmC) Alone				Non-medical Use of Opioids (NmO) Alone				Both NmC and NmO		
	Exposure to any ACEs	No ACEs Exposures	Standardized Difference, % (t-test)		Exposure to any ACEs	No ACEs Exposures	Standardized Difference, % (t-test)		Exposure to any ACEs	No ACEs Exposures	Standardized Difference, % (t-test)
	(N=5,471,539)	(N=3,236,341)			(N=5,614,724)	(N=3,283,488)			(N=5,848,632)	(N=3,313,102)	
State											
Arizona	(72.1%)	(72.1%)	0% (1.00)		(72.8%)	(72.8%)	0% (1.00)		(72.4%)	(72.4%)	0% (1.00)
Massachusetts	(27.9%)	(27.9%)	0% (1.00)		(27.2%)	(27.2%)	0% (1.00)		(27.6%)	(27.6%)	0% (1.00)
Metropolitan statistical area (MSA)											
Yes	(83.9%)	(83.9%)	0% (1.00)		(83.7%)	(83.7%)	0% (1.00)		(83.8%)	(83.8%)	0% (1.00)
No	(16.1%)	(16.1%)	0% (1.00)		(16.3%)	(16.3%)	0% (1.00)		(16.2%)	(16.2%)	0% (1.00)
Urban status											
Urban county	(95.2%)	(95.2%)	0% (1.00)		(95.1%)	(95.1%)	0% (1.00)		(95.1%)	(95.1%)	0% (1.00)
Rural county	(4.8%)	(4.8%)	0% (1.00)		(4.9%)	(4.9%)	0% (1.00)		(4.9%)	(4.9%)	0% (1.00)
Sex											
Male	(45.0%)	(45.0%)	0% (1.00)		(45.2%)	(45.2%)	0% (1.00)		(45.6%)	(45.6%)	0% (1.00)
Female	(55.0%)	(55.0%)	0% (1.00)		(54.8%)	(54.8%)	0% (1.00)		(54.4%)	(54.4%)	0% (1.00)
Race/ethnicity											
NH White	(72.7%)	(72.7%)	0% (1.00)		(72.5%)	(72.5%)	0% (1.00)		(72.5%)	(72.5%)	0% (1.00)
NH African American	(3.5%)	(3.5%)	0% (1.00)		(3.5%)	(3.5%)	0% (1.00)		(3.5%)	(3.5%)	0% (1.00)
NH Asian	(1.3%)	(1.3%)	0% (1.00)		(1.3%)	(1.3%)	0% (1.00)		(1.3%)	(1.3%)	0% (1.00)
NH American Indian or Alaskan Native	(4.0%)	(4.0%)	0% (1.00)		(4.0%)	(4.0%)	0% (1.00)		(4.1%)	(4.1%)	0% (1.00)
Hispanic	(14.9%)	(14.9%)	0% (1.00)		(15.0%)	(15.0%)	0% (1.00)		(15.0%)	(15.0%)	0% (1.00)
NH Other Race	(3.7%)	(3.7%)	0% (1.00)		(3.7%)	(3.7%)	0% (1.00)		(3.7%)	(3.7%)	0% (1.00)
Age, years											
18-34	(18.8%)	(18.8%)	0% (1.00)		(19.0%)	(19.0%)	0% (1.00)		(19.5%)	(19.5%)	0% (1.00)
35-54	(27.9%)	(27.9%)	0% (1.00)		(28.1%)	(28.1%)	0% (1.00)		(28.2%)	(28.2%)	0% (1.00)
55 or older	(53.3%)	(53.3%)	0% (1.00)		(52.9%)	(52.9%)	0% (1.00)		(52.3%)	(52.3%)	0% (1.00)

	Respondents Matched on Exposure to 2+ ACEs
	Respondents Who Reported Whether They Used

	**Non-medical Use of Cannabis (NmC) Alone**				**Non-medical Use of Opioids (NmO) Alone**				**Both NmC and NmO**		
	Exposure to 2+ ACEs	No ACEs Exposures	Standardized Difference, % (t-test)		Exposure to 2+ ACEs	No ACEs Exposures	Standardized Difference, % (t-test)		Exposure to 2+ ACEs	No ACEs Exposures	Standardized Difference, % (t-test)
	(N=4,111,556)	(N=4,592,120)			(N=4,229,049)	(N=4,664,956)			(N=4,433,575)	(N=4,724,944)	
State											
Arizona	(70.1%)	(70.1%)	0% (1.00)		(70.9%)	(70.9%)	0% (1.00)		(70.5%)	(70.5%)	0% (1.00)
Massachusetts	(29.9%)	(29.9%)	0% (1.00)		(29.1%)	(29.1%)	0% (1.00)		(29.5%)	(29.5%)	0% (1.00)
Metropolitan statistical area (MSA)											
Yes	(84.8%)	(84.8%)	0% (1.00)		(84.6%)	(84.6%)	0% (1.00)		(84.8%)	(84.8%)	0% (1.00)
No	(15.2%)	(15.2%)	0% (1.00)		(15.4%)	(15.4%)	0% (1.00)		(15.2%)	(15.2%)	0% (1.00)
Urban status											
Urban county	(95.2%)	(95.2%)	0% (1.00)		(95.1%)	(95.1%)	0% (1.00)		(95.1%)	(95.1%)	0% (1.00)
Rural county	(4.8%)	(4.8%)	0% (1.00)		(4.9%)	(4.9%)	0% (1.00)		(4.9%)	(4.9%)	0% (1.00)
Sex											
Male	(40.4%)	(40.4%)	0% (1.00)		(40.7%)	(40.7%)	0% (1.00)		(41.2%)	(41.2%)	0% (1.00)
Female	(59.6%)	(59.6%)	0% (1.00)		(59.3%)	(59.3%)	0% (1.00)		(58.8%)	(58.8%)	0% (1.00)
Race/ethnicity											
NH White	(71.3%)	(71.3%)	0% (1.00)		(71.2%)	(71.2%)	0% (1.00)		(71.1%)	(71.1%)	0% (1.00)
NH African American	(3.7%)	(3.7%)	0% (1.00)		(3.7%)	(3.7%)	0% (1.00)		(3.7%)	(3.7%)	0% (1.00)
NH Asian	(1.1%)	(1.1%)	0% (1.00)		(1.1%)	(1.1%)	0% (1.00)		(1.1%)	(1.1%)	0% (1.00)
NH American Indian or Alaskan Native	(4.0%)	(4.0%)	0% (1.00)		(4.1%)	(4.1%)	0% (1.00)		(4.1%)	(4.1%)	0% (1.00)
Hispanic	(15.5%)	(15.5%)	0% (1.00)		(15.5%)	(15.5%)	0% (1.00)		(15.6%)	(15.6%)	0% (1.00)
NH Other Race	(4.3%)	(4.3%)	0% (1.00)		(4.4%)	(4.4%)	0% (1.00)		(4.4%)	(4.4%)	0% (1.00)
Age, years											
18-34	(20.6%)	(20.6%)	0% (1.00)		(20.8%)	(20.8%)	0% (1.00)		(21.4%)	(21.4%)	0% (1.00)
35-54	(30.3%)	(30.3%)	0% (1.00)		(30.5%)	(30.5%)	0% (1.00)		(30.6%)	(30.6%)	0% (1.00)
55 or older	(49.1%)	(49.1%)	0% (1.00)		(48.7%)	(48.7%)	0% (1.00)		(48.0%)	(48.0%)	0% (1.00)

Abbreviations: ACEs = adverse childhood experiences

For every substance use outcome, we used propensity score methods to better isolate the exposure effect and to match persons with ACEs exposure to persons without ACEs on those characteristics that were unlikely affected by ACEs exposures, including location (state, urbanicity, MSA) and demographics (sex, race/ethnicity, age).

Table S2: ACEs Prevalence Among Adults Who Reported the Non-medical Use of Cannabis or Opioids and the Resulting Adverse Health Effects in Arizona and Massachusetts, 2019-2020.

	**Substance use**											
	**Non-medical cannabis (NmC) alone**				**Non-medical opioids (NmO) alone**				**Both substances**			
	**Yes**	**No**	**Difference**		**Yes**	**No**	**Difference**		**Yes**	**No**	**Difference**	
	**(95% CI)**	**(95% CI)**	**%**	**P Value**	**(95% CI)**	**(95% CI)**	**%**	**P Value**	**(95% CI)**	**(95% CI)**	**%**	**P Value**
**Respondents, N**	1,565,367	7,191,888	NA[a]	NA[a]	515,525	8,435,553	NA[a]	NA[a]	222,731	8,987,498	NA[a]	NA[a]
	(1,482,988 - 1,647,747)	(7,077,685 - 7,306,092)			(477,109 - 553,940)	(8,320,615 - 8,550,491)			(191,039 - 254,423)	(8,872,072 - 9,102,924)		
**Prevalence, %**												
**Any ACEs**	81.47	58.89	22.58	<.001	76.21	62.40	13.81	<.001	91.13	63.23	27.90	<.001
	(79.50 - 83.44)	(57.75 - 60.02)	(20.31- 24.85)		(73.21 - 79.21)	(61.35 - 63.46)	(10.58- 17.04)		(88.14 - 94.13)	(62.22 - 64.24)	(24.33- 31.47)	
**2+ ACEs**	66.74	42.77	23.97	<.001	62.54	46.46	16.08	<.001	80.97	47.39	33.58	<.001
	(64.19 - 69.28)	(41.62 - 43.93)	(21.15- 26.77)		(59.03 - 66.06)	(45.35 - 47.56)	(12.36- 19.82)		(75.52 - 86.41)	(46.33 - 48.46)	(27.98- 39.17)	
	**Adverse effects to substances**											
	**Non-medical cannabis (NmC) alone**				**Non-medical opioids (NmO) alone**				**Both substances**			
	**Yes**	**No**	**Difference**		**Yes**	**No**	**Difference**		**Yes**	**No**	**Difference**	
	**(95% CI)**	**(95% CI)**	**%**	**P Value**	**(95% CI)**	**(95% CI)**	**%**	**P Value**	**(95% CI)**	**(95% CI)**	**%**	**P Value**
**Respondents, N**	78,797	1,469,017	NA[a]	NA[a]	67,895	390,704	NA[a]	NA[a]	80,159	142,386	NA[a]	NA[a]
	(51,576 - 106,018)	(1,418,220 - 1,519,814)			(53,128 - 82,661)	(369,401 - 412,007)			(63,060 - 97,259)	(124,034 - 160,738)		
**Prevalence, %**												
**Any ACEs**	91.62	80.74	10.88	0.001	87.39	75.05	12.34	0.003	92.74	90.21	2.53	0.422
	(86.09 - 97.14)	(78.70 - 82.79)	(4.72- 17.03)		(78.12 - 100.81)	(51.07 - 82.97)	(4.81- 20.37)		(88.97 - 96.50)	(86.10 - 94.33)	(-3.66-8.71)	
**2+ ACEs**	89.42	65.25	24.17	<.001	82.08	59.54	22.54	<.001	84.41	79.00	5.41	0.353
	(83.66 - 95.18)	(62.64 - 67.87)	(17.33- 31.01)		(74.08 - 90.07)	(55.50 - 63.57)	(13.34- 32.73)		(74.82 - 94.01)	(72.31 - 85.69)	(-6.05- 16.87)	

Abbreviations: ACEs = adverse childhood experiences; CI = confidence interval.

All estimates in this table are weighted.

[a] Not applicable.

Table S3: Non-medical Use of Cannabis and Opioids in Arizona in 2020: Relative Risks and Population Attributable Fraction for the Actual ACEs Compared to Predicted ACEs Exposures.

	Substance use, actual ACEs					
	Non-medical cannabis (NmC) (past year use)			Non-medical opioids (NmO) (past year use)		
	Unadjusted RR (95% CI)	Adjustd RR [a] (95% CI)	PAF [b] (95% CI)	Unadjusted RR (95% CI)	Adjustd RR [a] (95% CI)	PAF [b] (95% CI)
Any ACEs	2.49	2.15	33.04	1.65	1.68	22.21
	(1.79 - 3.46)	(1.52 - 3.06)	(17.06-45.94)	(1.28 - 2.14)	(1.30 - 2.16)	(10.62-32.29)
2+ ACEs	2.37	2.07	30.60	1.67	1.66	22.97
	(1.79 - 3.13)	(1.52 - 2.81)	(17.43-41.67)	(1.33 - 2.10)	(1.31 - 2.09)	(11.62-31.10)
Number of observations	2,499,247	2,499,247	2,499,247	2,836,937	2,836,937	2,836,937

	Substance use, predicted ACEs					
	Non-medical cannabis (NmC) (past year use)			Non-medical opioids (NmO) (past year use)		
	Unadjusted RR (95% CI)	Adjustd RR [a] (95% CI)	PAF [b] (95% CI)	Unadjusted RR (95% CI)	Adjustd RR [a] (95% CI)	PAF [b] (95% CI)
Any ACEs	2.15	1.94	24.52	1.54	1.55	16.05
	(1.62 - 2.86)	(1.43 - 2.63)	(12.26-35.06)	(1.22 - 1.94)	(1.23 - 1.95)	(6.94-24.26)
2+ ACEs	2.37	2.07	30.60	1.67	1.66	22.97
	(1.79 - 3.13)	(1.52 - 2.81)	(17.43-41.67)	(1.33 - 2.10)	(1.31 - 2.09)	(11.62-31.10)
Number of observations	2,499,247	2,499,247	2,499,247	2,836,937	2,836,937	2,836,937

	Adverse health effects, actual ACEs					
	Non-medical cannabis (NmC) (past 6 months use)			Non-medical opioids (NmO) (past year use)		
	Unadjusted RR (90% CI)	Adjustd RR [a] (90% CI)	PAF [b] (90% CI)	Unadjusted RR (90% CI)	Adjustd RR [a] (90% CI)	PAF [b] (90% CI)
Any ACEs	1.09	1.14	NA	2.48	2.27	37.70
	(0.29 - 4.03)	(0.24 - 5.27)		(1.14 - 5.38)	(1.14 - 4.52)	(2.88-60.03)
2+ ACEs	1.63	1.91	31.05	2.66	2.05	34.60
	(0.59 - 4.54)	(0.50 - 7.36)	(-55.65-69.50)	(1.38 - 5.12)	(0.99 - 4.25)	(-4.36-59.01)
Number of observations	176,660	176,660	176,660	218,829	218,829	218,829

	Adverse health effects, predicted ACEs					
	Non-medical cannabis (NmC) (past 6 months use)			Non-medical opioids (NmO) (past year use)		
	Unadjusted RR (90% CI)	Adjustd RR [a] (90% CI)	PAF [b] (90% CI)	Unadjusted RR (90% CI)	Adjustd RR [a] (90% CI)	PAF [b] (90% CI)
Any ACEs	1.34	1.52	NA	2.01	1.53	18.79
	(0.43 - 4.20)	(0.45 - 5.14)		(1.04 - 3.89)	(0.79 - 2.96)	(-14.83-42.57)
2+ ACEs	1.63	1.91	31.05	2.66	2.05	34.60
	(0.59 - 4.54)	(0.50 - 7.36)	(-55.65-69.50)	(1.38 - 5.12)	(0.99 - 4.25)	(-4.36-59.01)
Number of observations	176,660	176,660	176,660	218,829	218,829	218,829

Abbreviations: ACEs = adverse childhood experiences; RR = relative risks; PAF = population attributable fractions

All estimates in this table are weighted. For every substance use outcome, we used propensity score methods to better isolate the exposure effect and to match persons with ACEs exposure to persons without ACEs on those characteristics that were unlikely affected by ACEs exposures, including location (state, urbanicity, MSA) and demographics (sex, race/ethnicity, age).

[a] Adjusted for respondent's demographic characteristics (sex, race/ethnicity, age), socio-economic characteristics (annual household income from all sources, education, employment, marital status, home ownership), access to healthcare (access to a personal healthcare provider, availability of any healthcare coverage), and location (metropolitan statistical area and urbanicity).

[b] In this table, we reported PAFs for the same outcome/ACEs combinations as in Table 3 to make results comparable across specifications. For instance, in this table PAFs for NmC AHE and any ACEs are not reported, since they were omitted in Table 3.
